# The memory of hunger: developmental plasticity of dietary selectivity in the European starling, *Sturnus vulgaris*

**DOI:** 10.1016/j.anbehav.2014.02.025

**Published:** 2014-05

**Authors:** Louise Bloxham, Melissa Bateson, Thomas Bedford, Ben Brilot, Daniel Nettle

**Affiliations:** a Centre for Behaviour and Evolution & Institute of Neuroscience, Newcastle University, U.K.; b School of Biological Sciences, Plymouth University, Plymouth, U.K.

**Keywords:** developmental plasticity, developmental stress, dietary cognition, nestling competition, starling

## Abstract

The decision to consume toxic prey is a trade-off between the benefits of obtaining nutrients and the costs of ingesting toxins. This trade-off is affected by current state: animals will consume more toxic prey if they are food deprived. However, whether the trade-off is affected by developmental history is currently unknown. We studied the decision to eat quinine-injected mealworms in adult starling siblings that had been exposed to either high or low levels of food competition as chicks, via a brood size manipulation. At the time of our experiments, the two groups of birds did not differ in size, body weight or current environment. Each bird was presented with the toxic prey while living on a high-quality diet and a low-quality diet. We found an effect of diet, with birds consuming more toxic prey while on the low-quality diet, and also of developmental history, with birds from the high-competition brood size treatment eating more toxic prey than their low-competition siblings. The effects of brood size treatment were not completely mediated by early growth, although we did find evidence that early growth affected toxic prey consumption independently of brood size treatment. We discuss our results in relation to adaptive developmental plasticity and the developmental origins of behavioural variation.

## Highlights

• Whether to eat toxic prey is a trade-off between getting nutrients and toxicity.
• Starlings faced either high or low food competition in early life.
• Birds that had faced high early competition ate more toxic prey as adults.
• Birds that grew poorly as chicks ate more toxic prey as adults.
• Sizes and weights of adult birds were not related to their early competition.

Animals face constant decisions about what to eat and what not to eat. While some items are never worth eating, there are many cases where the decision to eat or not should depend on the environment and the individual's current state (Kokko, Mappes, & Lindström, 2003; Sih & Christensen, 2001; Stephens & Krebs, 1986). For example, many potential prey available to birds in the wild are chemically defended, and so contain toxins that will be harmful in the long term or if eaten in excess (Eisner, Eisner, & Siegler, 2005; Eisner & Meinwald, 1966). However, such prey also contain valuable nutrients. In such cases, having lower energy reserves or poorer foraging prospects shifts the balance of costs and benefits in favour of consumption (Stephens & Krebs, 1986). European starlings whose body masses have been experimentally reduced become more willing to eat prey items that have been injected with quinine, which is toxic to birds in high doses (Barnett, Bateson, & Rowe, 2007; Barnett, Skelhorn, Bateson, & Rowe, 2012). These previous results show that current energetic state affects dietary decisions; in the current study, we considered the possible role of early developmental history in programming individuals' dietary selectivity.

There is evidence from rodent models that developmental history might influence adult dietary decisions. In particular, early food restriction induces an increased drive to obtain and consume food in adulthood (Qasem et al., 2012). Rats, *Rattus norvegicus*, whose mothers are fed restricted or low-protein diets in the perinatal period become hyperphagic, and this hyperphagia endures well beyond weaning (Coupé, Grit, Darmaun, & Parnet, 2009; Orozco-Sólis et al., 2009; Qasem et al., 2012; Vickers, Breier, Cutfield, Hofman, & Gluckman, 2000). The hyperphagia is particularly pronounced when the diet available in adulthood is of high quality (Vickers et al., 2000). However, no study has yet investigated how developmental history might influence dietary selectivity, in particular the point at which an animal will reject a foodstuff that contains both nutrients and toxins, rather than simply the amount of food consumed.

For passerine birds, a powerful, ecologically valid method for inducing early-life food competition, and hence lowering early-life food availability, is manipulation of brood size within the species' natural range of variation (Gil, Bulmer, Celis, & López-Rull, 2008; Naguib, Riebel, Marzal, & Gil, 2004; Verhulst, Holveck, & Riebel, 2006). As broods become larger, parents are unable to compensate fully by increasing the food supply, and chicks show poorer growth and markers of increased developmental stress (Naguib et al., 2004; Nettle, Monaghan, Boner, Gillespie, & Bateson, 2013; Wright & Cuthill, 1990). Being raised in a large brood has long-term effects on fitness and on various aspects of adult behaviour (Naguib & Gil, 2005; Naguib, Nemitz, & Gil, 2006; Riebel, Naguib, & Gil, 2009; Riebel, Spierings, Holveck, & Verhulst, 2012). While some of these effects are simply deleterious, others may represent adaptive responses to the conditions in which the individuals find themselves (Monaghan, 2008). For example, being raised in a large brood has been shown to make adult great tits, *Parus major*, more exploratory and more aggressive (Carere, Drent, Koolhaas, & Groothuis, 2005; see also Zimmer, Boogert, & Spencer, 2013 for related results). This could be an adaptive phenotype for conditions in which food will be scarce.

In the current study, we used brood size manipulation to alter early-life food competition in wild European starlings. We then reared the study birds in captivity and kept them in uniform conditions to adulthood, whereupon we studied their willingness to eat mealworms, *Tenebrio molitor*, injected with small amounts of toxic quinine. Quinine injection of mealworms has been widely used to study dietary selectivity in starlings. They rapidly learn to reject the toxic prey, but there is variation within and between birds in how many are rejected (Barnett et al., 2007, 2012; Chatelain, Halpin, & Rowe, 2013; Halpin, Skelhorn, & Rowe, 2013; Skelhorn & Rowe, 2006, 2007). To control for genetic effects, we used siblings assigned through cross-fostering to divergent brood sizes. We predicted that birds raised in large broods would be more willing than their siblings to consume the toxic mealworms. We also predicted that they might be hyperphagic overall, and to assess this, we recorded their ad libitum food consumption each day during the experiment. In addition, we investigated the effects of current energetic state on dietary selectivity, by performing our experiment while the birds were living under two different ad libitum dietary regimes, one of high quality and the other of low quality. In line with previous research (Barnett et al., 2007, 2012), we predicted that birds whose current diet was poor would be more willing to consume toxic prey. We also predicted that there might be interactions between current energetic state and developmental history, with birds that had experienced early-life food competition being more sensitive to variations in the quality of the current diet.

## Methods

### Ethical Note

Our study adhered to the ASAB/ABS Guidelines for the Use of Animals in Research, and was approved by the local ethical review committee at Newcastle University. It was completed under U.K. Home Office project licence number PPL 60/4073, and removal of starlings from the wild was authorized by Natural England (licence number 20121066). All fieldwork was carried out with the permission of landowners and invasiveness of field research was minimized. During cross-fostering, all chicks that hatched were given a nest and all parents that hatched chicks were given at least two chicks to raise.

### Subjects and Housing

Subjects were 31 European starlings taken from colonies on five farms in Northumberland, U.K. The birds had been cross-fostered shortly after hatching and subject to brood size manipulation (see below). They were brought into captivity 2 weeks after hatching and subsequently hand-reared. Birds were approximately 10 months old at the time of the experiments reported here. After completion of the experiments, the birds were permanently housed in an outdoor aviary at a zoo. During the period from hatching until the experiments reported here, three birds died in total: two nestlings were abandoned by their parents within 5 days of hatching, and one bird died after fledging, of unknown causes.

Prior to the start of the experiment, birds were group-housed in indoor aviaries enriched with water baths, perching ropes and suspended cardboard boxes as cover. For the experiment, birds were taken into the laboratory in groups of eight, and individually housed in wire-mesh cages measuring 45 × 75 cm and 45 cm high fitted with shelter, two wooden perches and two water bottles as well as bowls of water for bathing. The birds were maintained under a 13:11 h light:dark cycle with a temperature of 18 °C and 40% humidity. Birds were visually isolated from one another during the experimental sessions, but not for the remainder of the day. They were never acoustically isolated.

### Brood Size Manipulation

We have described the brood size manipulation in more detail elsewhere (Nettle et al., 2013). On posthatching day 3 (D3, where hatching is D1), quartets of focal siblings were removed from their natal nests. From each quartet, two chicks were cross-fostered to a host nest where they were the only chicks (the low-competition or LC treatment), while the other two were cross-fostered to a host nest that they shared with five other chicks of the same age (high-competition or HC treatment). Nonfocal competitors in the HC nests were also not in their natal nests. The size of the brood was the main difference between our treatments, but, additionally, HC birds were with nonsiblings and LC birds were not. However, there is no evidence in starlings of any effect of being with nonkin in the nest on developmental parameters (Smith & Wettermark, 1995). Thus, this kind of design is generally considered a clean manipulation of nestling competition (Gil et al., 2008; Wright & Cuthill, 1990).

Birds remained in these nests until they were brought into captivity on D15. Birds were transported to the laboratory by car (up to 60 min) in covered buckets containing nesting material. Once in captivity, the genetic families were recomposed in buckets and the birds hand-reared until fledging around D21 (for details of hand-rearing methods see Feenders & Bateson, 2011). From fledging until the time of the study, each aviary contained four complete families, and thus approximately equal numbers of HC and LC birds.

Brood size treatment had marked effects on growth rate, but not final size. As reported in our previous paper (Nettle et al., 2013), HC birds (*N* = 15) were significantly lighter than their LC siblings (*N* = 16) at D7, D11 and D15, but caught up after D15. At D20 and after independence their weights were not significantly different, and neither were their skeletal sizes as indicated by tarsus length at D15. There was greater variation in growth between the HC birds than the LC birds, with some HC individuals growing as fast as the LC mean, whereas others were slowed well beyond the range of LC variation (Nettle et al., 2013). To capture variation in early growth both between the two treatment groups and between individuals within a treatment group, we used weight on D11. D11 is towards the end of the period of linear growth, and at this time, fast-growing birds had reached essentially their adult weights, whereas the slowest-growing bird was still around 30 g lighter than its adult weight. D11 was the time point where the mean LC and HC weights differed most strongly, and the variance among the HC birds was largest.

### Ad Libitum Diets

Experimental sessions were held each morning, and no food other than experimental prey was available during the sessions. Ad libitum food was available at all other times. There were two different ad libitum diet regimes, provided in counterbalanced order across birds. Optimal diets for starlings are high in animal protein, and they also prefer ripe fruit when available. They can, however, switch to lower-valued plant foods when necessary (Feare, 1984). Thus, under our low-quality diet, birds received each day 30 g of commercial chick starter crumb, a grain-based diet containing 18.9% protein of vegetable origin. Under the high-quality diet, birds received each day 20 g of chick crumb plus 10 g of Orlux, a mixture of dried insects and crustaceans, ant eggs and ant larvae (29% protein), and one-eighth of an apple.

### Body Weight

Prior to the experimental session each day, birds were weighed remotely using a wooden perch secured to an electronic balance. To encourage them to perch on the balance, it was baited with two mealworm larvae (low-quality diet) or six mealworm larvae (high-quality diet). Daily consumption of ad libitum food was measured for each bird each day by weighing remaining food each morning. Eight birds (four from each brood size treatment group) were missing daily food consumption data for one of the diet phases.

### Prey Delivery and Behavioural Recording

Our experimental methods were based on those of Barnett et al. (2007). Live mealworm larvae were used as prey. During experimental trials, the palatability of the mealworms was manipulated by injecting the mealworms intraorally with 0.02 ml of water (palatable prey) or 0.02 ml of 2% quinine sulphate solution (toxic prey). Each prey type was associated with a distinct colour cue (see below). Although quinine is toxic, it was used in very low concentrations identical to those used in previous studies (Barnett et al., 2007, 2012; Skelhorn & Rowe, 2006, 2007) and we observed no ill effects. Prey were presented on a petri dish (55 mm diameter) mounted on a plain white ceramic tile. They were delivered into the cage through a small hatch by an experimenter who then retreated behind a curtain. Subsequent behavioural recording was via video link. Any uneaten prey were removed from the cage at the end of each 1 min trial.

### Experimental Procedures

**Figure 1:**
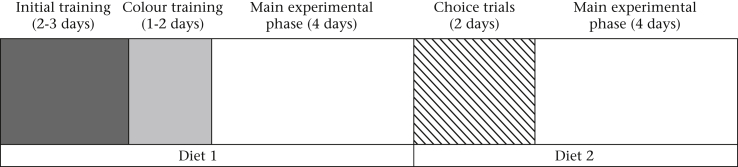
Timeline of the experiment for each group of birds.

The timeline of the experiment for each bird is summarized in Fig. 1. The birds were run in four successive groups of eight, with all members of a natal family run in the same group.

#### Initial training (2–3 days)

Birds were initially trained to eat uninjected mealworms from colourless petri dishes. Each day, birds received a training session consisting of 16 sequentially presented prey, with 4 min to attack each prey followed by an intertrial interval of 1 min. Birds advanced to the next day of training once they had successfully consumed six prey in succession within a day.

#### Colour training (1–2 days)

After initial training, the birds learned the association between a colour cue and prey type. Each prey type (palatable or toxic) was presented with a distinct colour background to the petri dish (pink or green). Colour pairing was counterbalanced across birds. On each day of colour training, birds were presented with 16 prey, eight palatable and eight toxic, in a pseudorandom order that ensured that there were never more than two prey of the same type in succession. Birds were given 1 min to attack the prey, followed by a 7 min intertrial interval. Colour training lasted 1 or 2 days, until each bird had consumed prey of both types.

#### Main experimental phase (4 + 4 days)

Once training was complete, birds underwent 8 days of experimental trials, 4 days under the low-quality diet and four under the high-quality diet. On each day, birds were presented sequentially with 16 prey on appropriately coloured backgrounds. Each prey was presented for 1 min with a 7 min intertrial interval. Again, eight prey were palatable and eight were toxic, in pseudorandom order. For each bird, we recorded the number of prey of each type eaten within the 1 min limit on each day. We also measured the bird's latency to attack the prey.

#### Choice trials (2 days)

In between the first and second diet blocks of the main experimental phase, birds were given 2 days of simultaneous choice trials. In these trials, two mealworms were presented simultaneously, one toxic and one palatable, with appropriate colour cues. Birds were allowed to attack only one mealworm before the tile was removed. The side on which each prey type was presented was alternated. Birds were presented with 16 choices per day, and we recorded the number of choices of each type of prey. The purpose of the choice trials was to establish that palatable prey were indeed preferred and the birds could tell the difference between the prey types.

### Consumption of Palatable Prey

All birds showed evidence of motivation to eat palatable prey during the experiment. Overall mean consumption of palatable prey was 31.83 of a possible 32 items under the low-protein diet, and 31.00 of a possible 32 under the high-protein diet. Birds also showed evidence of ability to discriminate palatable from toxic prey. For every bird, the number of palatable prey consumed over the 8 days of the main experimental phase was greater than or equal to the number of toxic prey. Birds' mean latencies to attack the mealworms were significantly shorter for palatable than unpalatable prey (paired *t* test: *t*_30_ = −4.503, *P* < 0.001; means + SE: palatable 2.761 + 0.937 s; unpalatable 8.336 + 0.937 s). In the choice trials, every bird chose the palatable prey more than half the time, and 26 birds had a significant preference (*P* < 0.05) for palatable over toxic prey on a binomial test. Those with a nonsignificant preference for palatable prey consisted of one LC and four HC.

### Data Analysis

Raw data from the study are available in the Supplementary material. Data were analysed using generalized linear mixed models in R (R Core Development Team, 2013), using the base statistical procedures and package lme4 (Bates, Maechler, Bolker, & Walker, 2013). R scripts are available on request. Model estimation was by maximum likelihood, and whether parameters differed significantly from 0 was determined by a *z* test with a critical value of *P* < 0.05. The text describes the main results relevant to the experimental hypotheses; full model output is provided in the Appendix.

The basic model for each outcome variable we studied included fixed effects for diet, brood size treatment and the interaction between diet and brood size treatment. In addition, since experience has shown that birds' behaviour changes as they become used to individual cages, the basic models also included a fixed effect of day of study. All models also included random intercepts for family (since quartets of birds were siblings) and bird (since the same individuals were measured for multiple days). For number of toxic prey, we also experimented with adding additional fixed effects of possible mediators, namely current weight during the study, and weight at D11, to the basic model, as described in the Results.

Models for body weight and ad libitum food consumption used a Gaussian error structure. Analysis of the residuals from the models indicated that this was an appropriate assumption. The number of toxic prey eaten each day was bounded by a maximum of eight, and non-normally distributed with many birds eating all eight on many days. Thus, for the analysis of this variable, we took the number of toxic prey rejected (i.e. eight minus the number eaten) and modelled it using negative binomial regression. This is a suitable approach for count data that are overdispersed relative to a Poisson distribution (Faraway, 2006). We also repeated the negative binomial regressions with Poisson regression, which yielded almost identical results (see Appendix).

## Results

### Body Weight

In the model for body weight, there was a significant effect of day of study, with weights increasing as the study progressed (*B* = 0.223, 95% CI 0.167 to 0.280, *z* = 7.762, *P* < 0.001). There was a nonsignificant trend for an effect of diet, with weights tending to be higher on the low-quality diet (*B* = −0.313, 95% CI −0.672 to 0.047, *z* = −1.705, *P* = 0.088). There was no effect of brood size treatment (*B* = −0.221, 95% CI −3.602 to 3.159, *z* = −0.128, *P* = 0.858). The overall mean body weights (+between-bird SD) were 74.828 + 4.213 g for the LC birds and 74.758 + 5.629 g for the HC birds. Nor was there a significant diet*brood size treatment interaction (*B* = 0.303, 95% CI −0.214 to 0.820, *z* = 1.150, *P* = 0.250).

### Ad Libitum Food Consumption

In the model for ad libitum food consumption during the experiment, there was a significant effect of day of study, with less consumed as the experiment proceeded (*B* = −0.396, 95% CI −0.536 to −0.255, *z* = −5.531, *P* < 0.001). There was also a significant effect of diet, with less food eaten under the high-quality diet (*B* = −2.063, 95% CI −2.957 to −1.170, *z* = −4.526, *P* < 0.001). However, there was no evidence of any significant difference between the LC birds (mean + between-bird SD 17.445 + 2.882 g) and the HC birds (mean + between-bird SD 18.108 + 3.836 g) in terms of daily ad libitum food consumption (*B* = 0.357, 95% CI −1.482 to 2.196, *z* = 0.381, *P* = 0.703). Nor was there any significant interaction between diet and brood size treatment (*B* = 0.625, 95% CI −0.637 to 1.887, *z* = 0.970, *P* = 0.332).

### Toxic Prey Consumption

**Figure 2:**
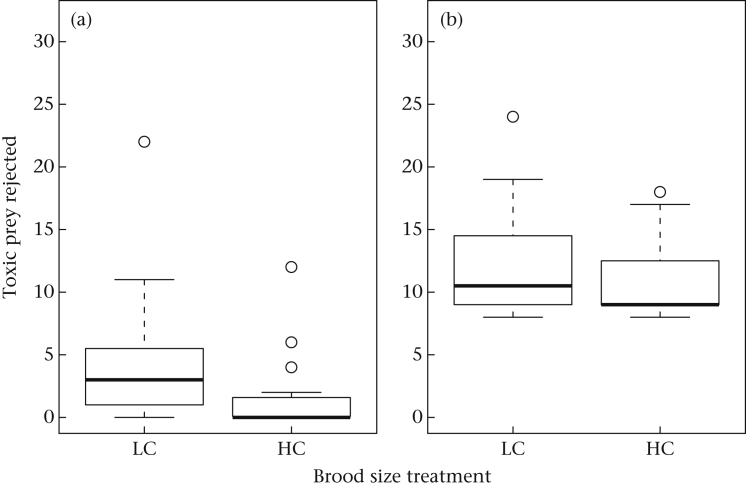
Box plot of the number of toxic prey rejected for birds from low-competition (LC) and high-competition (HC) brood size treatments, separately for (a) the low-quality diet and (b) the high-quality diet. The dark bars represent the median and the boxes the interquartile range. The whiskers represent the highest and lowest points within 1.5 times the interquartile range of the box. Circles are outliers.

In the basic model for toxic prey consumption, there was a significant effect of day of study, with birds rejecting more toxic prey as the experiment proceeded (*B* = 0.086, 95% CI 0.012 to 0.160, *z* = 2.280, *P* = 0.022), and a significant effect of diet, with more toxic prey rejected under the high-quality diet (*B* = 0.599, 95% CI 0.167 to 1.032, *z* = 2.718, *P* = 0.007). There was also a marginally significant effect of brood size treatment, with HC birds rejecting fewer toxic prey than LC birds (*B* = −0.875, 95% CI −1.730 to −0.019, *z* = −2.004, *P* = 0.045). The interaction between diet and brood size treatment was not significant (*B* = 0.450, 95% CI −0.256 to 1.157, *z* = 1.248, *P* = 0.212). Fig. 2 shows the total number of toxic prey rejected in each diet phase by brood size treatment. The effect of current diet was stronger than that of brood size treatment; the median number of toxic prey rejected over the 4 days was increased by 9 under the high-quality compared to the low-quality diet, whereas being of LC origin increased the median number of toxic prey rejected by 1.5 under the high-quality diet and 3 under the low-quality diet. When current body weight was added to the basic model, its effect was not significant (*B* = 0.006, 95% CI −0.069 to 0.080, *z* = 0.149, *P* = 0.881), and the effects of the other parameters were unchanged.

### Early Growth and Toxic Prey Consumption

To establish whether it was early growth that mediated the differences we observed between HC birds and LC birds, we added weight on D11 and its interactions with brood size treatment and diet to the basic model for toxic prey rejected. In this expanded model, there was a significant effect of weight on D11 (*B* = 0.212, 95% CI 0.075 to 0.349, *z* = 3.029, *P* < 0.001). The positive coefficient of weight on D11 means that birds that had been heavier at D11 rejected more toxic prey. There were also significant interactions between weight on D11 and diet (*B* = −0.113, 95% CI −0.177 to −0.049, *z* = −3.439, *P* < 0.001), and between weight on D11 and brood size treatment (*B* = −0.187, 95% CI −0.328 to −0.046, *z* = −2.601, *P* = 0.009). Even with weight on D11 and its two-way interactions in the model, the effect of brood size treatment remained significant (*B* = 13.392, 95% CI 3.08 to 23.700, *z* = 2.546, *P* = 0.011). Indeed, it was more strongly significant than in the basic model. Thus, the effects of brood size treatment were not completely mediated by early growth.

**Figure 3:**
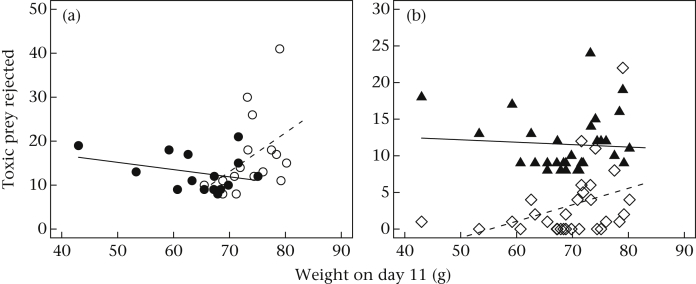
Effects of weight on D11, a measure of early growth, on toxic prey rejected during the main experimental phase. (a) Weight on D11 against total number of toxic prey rejected (of a possible 64) for the LC birds (open circles) and the HC birds (filled circles). (b) Weight on D11 against number of toxic prey consumed (of a possible 32) under the low-quality diet (open diamonds) and the high-quality diet (filled triangles). Note that each bird appears twice in this figure, once under each diet regime.

The interaction effects involving weight on D11 are visualized in Fig. 3. Fig. 3a plots number of toxic prey rejected over the whole of the main experimental phase against weight on D11, for the two brood size treatment groups. The positive relationship between weight on D11 and toxic prey rejected was restricted to the LC birds. All of the HC birds rejected relatively few toxic prey, and to the extent that there was any effect of weight on D11 within them, it was those HC birds that were relatively heavier on D11 that rejected the fewest. Fig. 3b plots toxic prey rejected under each of the two diets separately, against weight on D11. Here, the positive effect of weight on D11 on toxic prey rejection was evident only under the low-protein diet, and absent under the high-protein diet.

## Discussion

We studied consumption of toxic quinine-injected prey by adult starlings that been subjected to either high or low food competition as nestlings, under high- and low-quality current diets. We found main effects of current diet (birds ate fewer toxic prey when living on a high-quality diet), and of brood size treatment (birds raised in large broods ate more toxic prey than their siblings raised in small broods). The interaction between current diet and brood size treatment was not significant. There was clear evidence that the birds from both treatment groups could discriminate toxic from palatable prey and had a preference for palatable prey. Thus, it is likely that the effects of diet and brood size treatment reflect alterations in consumption decisions rather than alterations in the ability to differentiate toxic from palatable prey.

The substantial main effect of current diet adds to the considerable existing evidence that starlings modulate their consumption of protein-rich but toxic foods according to their current state (Barnett et al., 2007, 2012; Skelhorn & Rowe, 2007). Our results advance knowledge in this area by showing that it is not necessary to food-deprive starlings to increase consumption of toxic prey. It is sufficient to alter diet quality. Our high-quality diet differed from our low-quality diet in a number of ways, but the most salient difference may have been the absence of animal protein. Optimal starling diets contain high levels of animal protein (Feare, 1984), whereas our low-quality diet was grain-based. Thus, although the birds significantly increased their daily consumption of ad libitum food during the low-quality diet phase, they still would have been relatively protein-deprived. The increased consumption of toxic mealworms thus represents a strategic shift in the benefits of animal protein relative to the costs of toxin consumption.

The brood size treatment effect was smaller in magnitude than the effect of current diet, but none the less significant. It accords with our predictions made on the basis of the evidence of hyperphagia following early-life food restriction in rats (Coupé et al., 2009; Orozco-Sólis et al., 2009; Qasem et al., 2012; Vickers et al., 2000). It also confirms previous avian evidence that brood size manipulations can induce altered behavioural phenotypes enduring into adulthood (Carere et al., 2005; Riebel et al., 2009, 2012). Unlike the rat studies, though, our HC birds were not hyperphagic overall, since their consumption of daily ad libitum food was not significantly increased. Their increased consumption was restricted to the experimental prey. Mealworms are an extremely valued food for starlings, and thus the pattern is consistent with the observation in rats that the hyperphagia effects are particularly pronounced for very high-quality foods (Vickers et al., 2000). Our HC birds were no different from their LC siblings in terms of adult size or body weight. This is in contrast to the rat studies, in which animals subjected to early-life food deprivation were significantly lighter than control animals at the time of study (Qasem et al., 2012; Vickers et al., 2000). Thus, the rat evidence is consistent with the possibility that the enduring hyperphagia following early-life food restriction is driven by current energy reserves in adulthood. In our case, the animals appeared to retain a memory of early food competition even though their body weights were now equal.

We investigated the effects of early growth on toxic prey consumption using weight on D11 as a summary measure. Overall, being lighter on D11 predicted rejecting fewer (i.e. consuming more) toxic prey during the experiment. This was true under the low-quality diet in particular, suggesting that poor early growth makes individuals more sensitive to current nutritional deficits. However, although average weight on D11 was lower in the HC treatment group than the LC treatment group, weight on D11 did not mediate the brood size treatment effect. Indeed, the HC birds that rejected the fewest (i.e. consumed the most) toxic prey were actually those whose early growth had been relatively good. This suggests that the experience of nestling competition has phenotypic consequences above and beyond its direct effects on growth.

Overall, our results can be plausibly interpreted within the framework of adaptive developmental plasticity. It would be plausibly beneficial for animals facing high competition or low food availability to reduce their dietary selectivity, thus increasing the relative priority of getting food and decreasing the relative priority of other imperatives such as toxin avoidance. This principle would explain both the brood size treatment effect, and the main effect of poor early growth, on toxic prey consumption. However, we studied our birds nearly a year after the competition manipulation had finished. This raises the question of why, even if adopting an unselective dietary phenotype is adaptive for survival immediately after fledging where food competition has been high, the trait has to endure into adulthood when current environmental conditions no longer require it. It would appear advantageous to be able to switch off the phenotype as soon as body weights have equalized.

Why animals do not remain permanently plastic, and instead show lasting, stable effects of early conditions are subjects of theoretical debate (Dall, Houston, & McNamara, 2004; DeWitt, Sih, & Wilson, 1998; Frankenhuis & Panchanathan, 2011; Moran, 1992; Wolf, van Doorn, & Weissing, 2008). In this case, there are two possibilities. One is that the effects in adulthood are a nonadaptive hangover of a behavioural calibration that is strongly advantageous during the nestling period. The other is that the unselective phenotype remains adaptively beneficial into adulthood for birds that have experienced a poor start. This would be true if, for example, starlings that get a poor start in life tend to be disadvantaged in social competition as adults, because they are on average less vigorous or of lower quality. If this were the case, it would make adaptive sense to recalibrate their dietary decisions permanently, as an anticipatory adjustment to the competitive disadvantage that they will be likely to face as adults. Alternatively, the birds that faced early competition might have had poorer energetic reserves or robustness to food deprivation in some way that was not captured by current body weight. In birds, nestling competition (Verhulst et al., 2006) and early-life corticosterone exposure (Spencer & Verhulst, 2008) can increase adult metabolic rate. Thus, it is possible that our HC birds and birds with poor early growth have a greater metabolic requirement as adults. This could be relevant to their reduced food selectivity and increased sensitivity to current poor diet. However, it would predict increased food consumption overall, of which we found no evidence.

Perhaps the most striking feature of our results is that in their first year of life, the sibling pairs experienced only 12 days of divergent experience, with the rest of the time spent living in the same environment. This rather modest amount of differential exposure to competition, coming during the critical growth period, appears to have been sufficient to induce a different, long-lasting, behavioural phenotype. We may owe our ability to detect such differences in a modest sample to our use of a sibling design that controls for genetic variation. This allowed us to isolate the developmental influence from other sources of variation. Understanding how and why early conditions have such marked effects is important for evolutionary biologists studying phenotypic plasticity and the origins of individual differences. It is also potentially important in the quest to understand the developmental origins of food-related conditions such as obesity and metabolic syndrome in humans.

## Figures and Tables

**Table A1:** Model output for body weight and ad libitum food consumption during the experiment

Fixed effects	Body weight			Ad libitum food consumption		
	Parameter estimate	SE	*z*	Parameter estimate	SE	*z*
Day of study	0.223	0.029	7.762*	−0.396	0.072	−5.531*
Diet	−0.313	0.183	−1.705	−2.063	0.456	−4.526*
Brood size treatment	−0.221	1.725	−0.128	0.357	0.938	0.381
Diet*Brood size treatment	0.303	0.264	1.150	0.625	0.644	0.970
**Variances**						
Bird within family		22.765			5.567	
Family		0.000			1.846	
Residual		1.075			4.869	

*P < 0.05.

**Table A2:** Model output for the basic and expanded negative binomial models of number of toxic prey rejected

Fixed effects	Basic model			Expanded model		
	Parameter estimate	SE	*z*	Parameter estimate	SE	*z*
Day of study	0.086	0.038	2.280*	0.067	0.038	1.773
Diet	0.599	0.221	2.718*	9.114	2.487	3.665*
Brood size treatment	−0.875	0.436	−2.004*	13.392	5.259	2.546*
Diet*Brood size treatment	0.450	0.361	1.248	−1.003	0.523	1.915
Weight D11				0.212	0.070	3.029*
Weight D11*Diet				−0.113	0.033	−3.439*
Weight D11*Brood size treatment				−0.187	0.072	−2.601*
**Variances**						
Bird within family		0.754			0.665	
Family		0.440			0.218	
Residual		0.927			1.093	

*P < 0.05.

**Table A3:** Model output for basic and expanded Poisson models of number of toxic prey rejected

Fixed effects	Basic model			Expanded model		
	Parameter estimate	SE	*z*	Parameter estimate	SE	*z*
Day of study	0.065	0.026	2.469*	0.059	0.027	2.162*
Diet	0.615	0.148	4.149*	10.797	2.128	5.074*
Brood size treatment	−0.866	0.424	−2.044*	13.767	5.108	2.695*
Diet*Brood size treatment	0.459	0.269	1.709	−1.335	0.426	−3.135*
Weight D11				0.230	0.068	3.390*
Weight D11*Diet				−0.135	0.028	−4.809*
Weight D11*Brood size treatment				−0.189	0.070	−2.703*
**Variances**						
Bird within family		0.855			0.674	
Family		0.586			0.246	

*P < 0.05.

## Appendix Model output and supplementary analyses

This appendix provides the full output from the statistical models described in the Results, plus additional Poisson models for toxic prey rejection. Table A1 shows the model output for body weight and ad libitum food consumption during the experiment. Table A2 provides the output for the negative binomial models of toxic prey consumption during the experiment. It shows both the basic model, and the expanded model including weight at D11 and its interactions.

An alternative approach to negative binomial regression for right-skewed count data is Poisson regression. The Poisson regression model is simpler than negative binomial regression and is suitable when the variance in the outcome variable within each treatment group is approximately equal to the mean (Faraway, 2006). For toxic prey rejected in our experiment, the mean was 1.26 and the variance of the simple residuals from the main experimental model was 2.65. Thus, there was some evidence for overdispersion relative to a Poisson distribution, justifying our choice of negative binomial regression. However, to investigate whether our results were robust to choice of model structure, we also repeated the negative binomial analyses using Poisson regression. The output from the Poisson regression models is reproduced in Table A3 for comparison with Table A2. The inferences are identical and the parameter estimates are very similar to those obtained with the negative binomial regression.

## References

[bib1] Barnett C.A., Bateson M., Rowe C. (2007). State-dependent decision making: educated predators strategically trade off the costs and benefits of consuming aposematic prey. Behavioral Ecology.

[bib2] Barnett C.A., Skelhorn J., Bateson M., Rowe C. (2012). Educated predators make strategic decisions to eat defended prey according to their toxin content. Behavioral Ecology.

[bib3] Bates D., Maechler M., Bolker B.M., Walker S. (2013). lme4: Linear mixed-effects models using Eigen and S4 (Version 1.0-4). http://cran.r-project.org/package=lme4.

[bib4] Carere C., Drent P.J., Koolhaas J.M., Groothuis T.G.G. (2005). Epigenetic effects on personality traits: early food provisioning and sibling competition. Behaviour.

[bib5] Chatelain M., Halpin C.G., Rowe C. (2013). Ambient temperature influences birds' decisions to eat toxic prey. Animal Behaviour.

[bib6] Coupé B., Grit I., Darmaun D., Parnet P. (2009). The timing of “catch-up growth” affects metabolism and appetite regulation in male rats born with intrauterine growth restriction. American Journal of Physiology: Regulatory Integrative and Comparative Physiology.

[bib7] Dall S.R.X., Houston A.I., McNamara J.M. (2004). The behavioural ecology of personality: consistent individual differences from an adaptive perspective. Ecology Letters.

[bib8] DeWitt T.J., Sih A., Wilson D.S. (1998). Costs and limits of phenotypic plasticity. Trends in Ecology & Evolution.

[bib9] Eisner T., Eisner M., Siegler (2005). Secret weapons: Defences in insects, spiders, scorpions, and other many-legged creatures.

[bib10] Eisner T., Meinwald J. (1966). Defensive secretions of arthropods. Science.

[bib11] Faraway J.J. (2006). Extending the linear model with R: Generalized linear, mixed effects and non parametric regression models.

[bib12] Feare C. (1984). The Starling.

[bib13] Feenders G., Bateson M. (2011). Hand-rearing reduces fear of humans in European starlings, *Sturnus vulgaris*. PLoS ONE.

[bib14] Frankenhuis W.E., Panchanathan K. (2011). Balancing sampling and specialization: an adaptationist model of incremental development. Proceedings of the Royal Society B: Biological Sciences.

[bib15] Gil D., Bulmer E., Celis P., López-Rull I. (2008). Adaptive developmental plasticity in growing nestlings: sibling competition induces differential gape growth. Proceedings of the Royal Society B: Biological Sciences.

[bib16] Halpin C.G., Skelhorn J., Rowe C. (2013). Predators' decisions to eat defended prey depend on the size of undefended prey. Animal Behaviour.

[bib17] Kokko H., Mappes J., Lindström L. (2003). Alternative prey can change model-mimic dynamics between parasitism and mutualism. Ecology Letters.

[bib18] Monaghan P. (2008). Early growth conditions, phenotypic development and environmental change. Philosophical Transactions of the Royal Society B: Biological Sciences.

[bib19] Moran N.A. (1992). The evolutionary maintenance of alternative phenotypes. American Naturalist.

[bib20] Naguib M., Gil D. (2005). Transgenerational effects on body size caused by early developmental stress in zebra finches. Biology Letters.

[bib21] Naguib M., Nemitz A., Gil D. (2006). Maternal developmental stress reduces reproductive success of female offspring in zebra finches. Proceedings of the Royal Society B: Biological Sciences.

[bib22] Naguib M., Riebel K., Marzal A., Gil D. (2004). Nestling immunocompetence and testosterone covary with brood size in a songbird. Proceedings of the Royal Society B: Biological Sciences.

[bib23] Nettle D., Monaghan P., Boner W., Gillespie R., Bateson M. (2013). Bottom of the heap: having heavier competitors accelerates early-life telomere loss in the European starling, *Sturnus vulgaris*. PLoS One.

[bib24] Orozco-Sólis R., Lopes de Souza S., Barbosa Matos R.J., Grit I., Le Bloch J., Nguyen P. (2009). Perinatal undernutrition-induced obesity is independent of the developmental programming of feeding. Physiology and Behavior.

[bib25] Qasem R.J., Yablonski E., Li J., Tang H.M., Pontiggia L., D'Mello A.P. (2012). Elucidation of thrifty features in adult rats exposed to protein restriction during gestation and lactation. Physiology and Behavior.

[bib26] R Core Development Team (2013). R: A language and environment for statistical computing. http://www.r-project.org/.

[bib27] Riebel K., Naguib M., Gil D. (2009). Experimental manipulation of the rearing environment influences adult female zebra finch song preferences. Animal Behaviour.

[bib28] Riebel K., Spierings M.J., Holveck M.J., Verhulst S. (2012). Phenotypic plasticity of avian social-learning strategies. Animal Behaviour.

[bib29] Sih A., Christensen B. (2001). Optimal diet theory: when does it work, and when and why does it fail?. Animal Behaviour.

[bib30] Skelhorn J., Rowe C. (2006). Predator avoidance learning of prey with secreted or stored defences and the evolution of insect defences. Animal Behaviour.

[bib31] Skelhorn J., Rowe C. (2007). Predators' toxin burdens influence their strategic decisions to eat toxic prey. Current Biology.

[bib32] Smith H.G., Wettermark K.J. (1995). Heritability of nestling growth in cross-fostered European starlings *Sturnus vulgaris*. Genetics.

[bib33] Spencer K.A., Verhulst S. (2008). Post-natal exposure to corticosterone affects standard metabolic rate in the zebra finch (*Taeniopygia guttata*). General and Comparative Endocrinology.

[bib34] Stephens D.W., Krebs J.R. (1986). Foraging theory.

[bib35] Verhulst S., Holveck M.-J., Riebel K. (2006). Long-term effects of manipulated natal brood size on metabolic rate in zebra finches. Biology Letters.

[bib36] Vickers M.H., Breier B.H., Cutfield W.S., Hofman P.L., Gluckman P.D. (2000). Fetal origins of hyperphagia, obesity, and hypertension and postnatal amplification by hypercaloric nutrition. American Journal of Physiology: Endocrinology and Metabolism.

[bib37] Wolf M., van Doorn G.S., Weissing F.J. (2008). Evolutionary emergence of responsive and unresponsive personalities. Proceedings of the National Academy of Sciences of the United States of America.

[bib38] Wright J., Cuthill I. (1990). Biparental care: short-term manipulation of partner contribution and brood size in the starling, *Sturnus vulgaris*. Behavioral Ecology.

[bib39] Zimmer C., Boogert N.J., Spencer K.A. (2013). Developmental programming: cumulative effects of increased pre-hatching corticosterone levels and post-hatching unpredictable food availability on physiology and behaviour in adulthood. Hormones and Behavior.

